# Research Progress in Composite Materials for Photocatalytic Nitrogen Fixation

**DOI:** 10.3390/molecules28217277

**Published:** 2023-10-26

**Authors:** Cheng Zuo, Qian Su, Lei Yu

**Affiliations:** College of Chemistry & Chemical and Environmental Engineering, Weifang University, Weifang 261061, China; 17854270427@163.com (C.Z.); sqian316@wfu.edu.cn (Q.S.)

**Keywords:** nitrogen, ammonia synthesis, photocatalysis technology, photocatalysts

## Abstract

Ammonia is an essential component of modern chemical products and the building unit of natural life molecules. The Haber–Bosch (H-B) process is mainly used in the ammonia synthesis process in the industry. In this process, nitrogen and hydrogen react to produce ammonia with metal catalysts under high temperatures and pressure. However, the H-B process consumes a lot of energy and simultaneously emits greenhouse gases. In the “double carbon” effect, to promote the combination of photocatalytic technology and artificial nitrogen fixation, the development of green synthetic reactions has been widely discussed. Using an inexhaustible supply of sunlight as a power source, researchers have used photocatalysts to reduce nitrogen to ammonia, which is energy-dense and easy to store and transport. This process completes the conversion from light energy to chemical energy. At the same time, it achieves zero carbon emissions, reducing energy consumption and environmental pollution in industrial ammonia synthesis from the source. The application of photocatalytic technology in the nitrogen cycle has become one of the research hotspots in the new energy field. This article provides a classification of and an introduction to nitrogen-fixing photocatalysts reported in recent years and prospects the future development trends in this field.

## 1. Introduction

**Figure 1:**
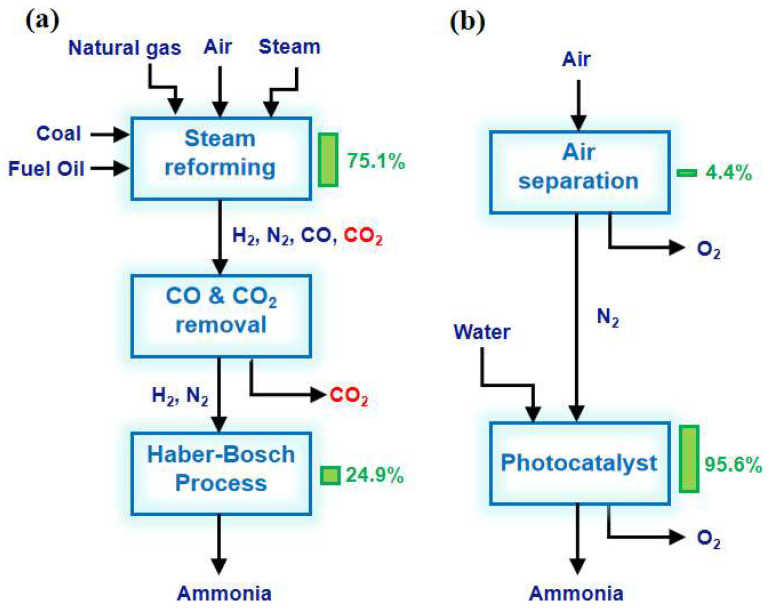
Energy efficiency analysis of (**a**) the H-B process and (**b**) photocatalytic nitrogen fixation for NH_3_ synthesis. The columns and numbers to the right of the block represent the share of the total energy input [4]. Copyright 2018, Cell Press.

With the rapid development of the global economy, energy sources and the environment are being irreversibly damaged, threatening the survival and development of humankind. It is urgent to find solutions to the energy crisis and environmental pollution. Ammonia is one of the most highly produced chemicals in the world [1]. The progress of production directly affects the energy structure and environmental issues. Currently, global NH_3_ production is approximately 170 million tons per year and highly relies on the traditional Haber–Bosch (H-B) process [2,3]. Industrial ammonia synthesis is usually carried out at high temperatures to improve the reaction rate and maintain optimal catalyst activity (Table 1). At the same time, the H-B process uses high pressure to overcome thermodynamic limitations and promote a rightward shift in reaction equilibrium, thereby improving conversion rates. Despite the harsh reaction conditions, the one-way conversion rate of synthetic NH_3_ can only reach 10–15%. In addition, the H-B process is powered by energy from the reforming or gasification of natural gas and fossil fuels using pressurized superheated steam. The H_2_ production process consumes about 75% of the energy input and produces half as much carbon dioxide as the entire process. The annual emissions of CO_2_ greenhouse gases from the entire H-B process amount to 300 million tons, accounting for approximately 1.6% of the global total emissions (Figure 1a) [4]. Therefore, finding a new substance to replace H_2_ as a proton source while overcoming harsh reaction conditions is an ideal method to reduce fossil energy consumption and CO_2_ emissions [5]. Photocatalytic nitrogen fixation technology utilizes renewable solar energy as the energy source to achieve the catalytic synthesis of NH_3_ from N_2_ and water under mild conditions. In addition, photocatalysis, capable of promoting thermodynamic non-spontaneous N_2_ reduction reactions, would be a green and sustainable alternative to the H-B process by continuously supplying electrons to activate adsorbed molecules to reduce N_2_ in synthesizing NH_3_ (Figure 1b).

In the nitrogen reduction reaction, the efficiency of photocatalytic ammonia synthesis remains low due to the inert structure of the N_2_ molecule, the difficulty in dissociating the N≡N bond, and the presence of high-energy intermediates (e.g., N_2_H) [6,7]. The low reaction efficiency severely limits the development and application of photocatalysis. Enhancing the reaction activity using efficient photocatalysts is the core of photocatalytic nitrogen fixation. In addition, the insolubility of nitrogen limits the gas–liquid contact of the non-homogeneous reaction system, which reduces the efficiency of the photocatalytic reaction. Therefore, establishing a new photocatalytic reaction system to improve the utilization of visible light and enhance the gas–liquid mass transfer ability is an essential means to realize the high efficiency and stability of photocatalytic nitrogen fixation reactions.

Currently, the research on photocatalysts for reducing nitrogen is mainly focused on improving the overall reaction efficiency, including light absorption, the separation and migration of photogenerated carriers, and the surface-catalyzed reaction. Several common photocatalytic materials, such as metal oxides, metal sulfides, bismuth halides, carbon-based materials, and MOFs, are presented in this paper. The semiconductor materials’ morphology, structure, and properties are analyzed to construct tunable catalytic systems. A structural morphology with a large specific surface area is prepared by changing the reaction conditions, precursor composition and ratio, and using other strategies to improve the contact chances between the catalyst and the reactants, increase the active sites, promote the adsorption and activation of N_2_, the rapid dissociation of the N≡N bond, as well as the reduction in the production of energetic intermediates. In addition, the semiconductor bandgap width was adjusted by introducing vacancies, constructing heterojunctions, and elemental doping to reduce the bandgap, realize the catalyst’s response to visible light, and improve light energy utilization [8,9,10].

In future research, we will analyze the mechanism of photocatalysts and reactions in combination with the density functional theory (DFT) and the feedback of the experimental results. We strive to create a set of reasonable and efficient photocatalytic reaction systems tailored for nitrogen fixation to achieve high energy utilization and excellent catalytic activity in the nitrogen fixation process.

## 2. Photocatalysts for Nitrogen Fixation

The photocatalyst is one of the critical factors determining whether the photocatalytic nitrogen fixation process can be carried out smoothly. So far, researchers have conducted systematic studies on designing efficient photocatalytic materials and developed numerous photocatalysts that could realize nitrogen reduction under mild conditions. Depending on the elemental composition, the materials used for nitrogen fixation reactions include metal oxides, metal sulfides, bismuth halides, carbon-based materials, and MOFs.

### 2.1. Metal Oxides

The use of metal oxides as materials for photocatalytic nitrogen fixation dates back to the pioneering work of Schrauzer and Guth in 1977 [11]. Their study successfully reduced nitrogen to ammonia and a small amount of hydrazine (N_2_H_4_) using Fe_2_O_3_-doped TiO_2_ as the catalyst and UV light and water as the light and proton sources, respectively. By adjusting the Fe doping amount, the experiment showed that TiO_2_ containing 0.2% Fe had the best ammonia production rate. Inspired by this, Augugliaro et al. [12] prepared a series of Fe-doped TiO_2_ using co-precipitation and impregnation techniques to investigate the nitrogen fixation activity of the samples in a continuous fixed-bed reactor, to analyze the roles of OH and Fe^3+^ on the surface of the catalysts in the reaction process, and to probe the reaction nature of photocatalysis. Radford et al. [13] synthesized Fe-doped anatase and rutile TiO_2_ by metal vaporization. It was found that the undoped samples could not drive the nitrogen reduction reaction, whereas the Fe-doped samples were endowed with catalytic activity to drive the reaction, and the Fe-doped anatase had more negative flat-band potential energy, thus having higher activity. Based on in-depth investigation, the mechanism of Fe in the photocatalytic reaction was mainly reflected in two aspects: On the one hand, the appropriate amount of Fe doping could capture photogenerated electrons and inhibit the recombination of photogenerated carriers [14]. On the other hand, Fe doping could generate oxygen vacancies and corresponding defect energy levels, and the high spin state Fe(III) prompts Fe 3d electrons to feedback to the N 1πg* orbitals to activate the adsorbed nitrogen molecules [15]. Other transition metals such as Ru, Co, Mo, and Ni have been shown to contribute to the catalytic performance when introduced as dopants into TiO_2_ [16,17]. In addition to element doping, constructing heterostructures to improve the separation and transportation of photogenerated charges is also an effective means to enhance photocatalytic activity and stability. For example, TiO_2_/Cu_7_S_4_ composites were loaded onto copper mesh by hydrothermal and calcination methods, forming an S-scheme heterojunction at the interface [18]. The calcination treatment increases the specific surface area and surface defects of the photocatalyst. The rich oxygen vacancies and S-scheme heterostructures of photocatalysts accelerate the separation and transport of photogenerated carriers, resulting in a strong redox ability of photocatalysts. Under visible light, the yield of NH_3_ synthesized by the OV-TiO_2_@Cu_7_S_4_ photocatalyst reached 133.42 μmol·cm^−2^·h^−1^, which is 5.2 and 2.2 times that of pure TiO_2_ and Cu_7_S_4_, respectively.

**Figure 2:**
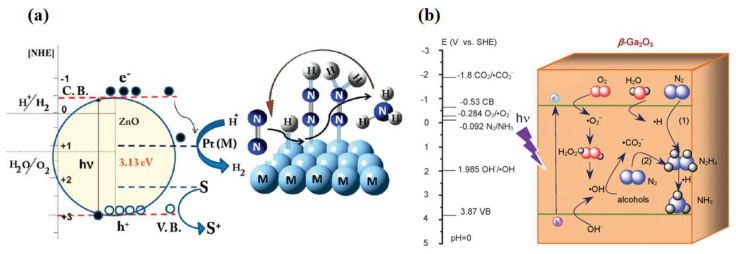
(**a**) Schematic representation of simultaneous hydrogen production and nitrogen reduction on Pt-doped ZnO [24]. (**b**) Possible direct and indirect electron transfer pathways on β-Ga_2_O_3_ photocatalysts [26]. Copyright 2017, Elsevier.

In addition to TiO_2_, other metal oxides such as iron oxide (Fe_2_O_3_) [19,20], bismuth oxide (BiO) [21], tungsten oxide (WO_3_) [22,23], zinc oxide (ZnO) [24,25], and gallium oxide (Ga_2_O_3_) [26,27] have been used as candidates for photocatalytic nitrogen fixation materials. Khader et al. [19] used α-Fe_2_O_3_ partially reduced to Fe_3_O_4_ in the presence of 3–5% divalent iron ions in the catalyst, and ammonia production was detected in the catalyst slurry by UV irradiation. Fe_2_O_3_ was shown to be an effective photocatalyst for nitrogen reduction, and its narrow bandgap feature enabled response to visible light [28]. Wang et al. [11] used a simple hydrothermal synthesis method to prepare low-valent Bi^2+^ containing BiO materials for photocatalytic nitrogen fixation. As shown in Figure 1a, unlike ordinary Bi^3+^, Bi^2+^ in BiO has empty 6d orbitals that accept electrons from N_2_ and provide high-quality chemisorption and activation centers. N_2_ was activated by three aligned Bi atoms by supplying electrons to the 6d orbitals of Bi and accepting lone pairs of electrons from the three Bi atoms into their empty antibonding orbitals (σ*2p_x_, π*2p_y_, and π*2p_z_), generating a 1N_2_-3Bi(II) side-pair bonding structure, which significantly weakened the N≡N bond and accelerated the photocatalytic NRR process. Hao et al. [29] employed nanostructured Bi_2_MoO_6_ crystals as a novel photocatalyst for synthesizing ammonia from air and water molecules without adding any sacrificial agent. The significantly improved photocatalytic nitrogen fixation performance (1.3 mmol·g_cat_^−1^·h^−1^) was mainly attributed to the ligand-unsaturated Mo atoms exposed at the edges of the MoO_6_ polyhedra becoming the active centers to promote the chemisorption activation process of N_2_. Introducing oxygen vacancies or noble metals on the surface to construct active centers was the key to improving photocatalytic activity for the WO_3_ and ZnO. According to Hou et al. [22], the grain boundaries (GBs) in nanoporous WO_3_ were induced to produce abundant surface defects under light, which were able to modulate the energy band structure, enhance the W-O covalency, and drive the photogenerated electron transfer to adsorbed N_2_. This significantly enhanced the nitrogen-fixing activity of WO_3_-600. Janet et al. [24] used wet etching and chemical precipitation to synthesize Pt-loaded ZnO with increased active centers resulting in a reactive ammonia yield of 86 μmol·g_cat_^−1^·h^−1^ at ambient temperature and pressure (Figure 2a). Zhao et al. [26] used uniformly stabilized mesoporous β-Ga_2_O_3_ nanorods as photocatalysts for photocatalytic nitrogen fixation under UV light irradiation (λ = 254 nm). The broad bandgap of the synthesized β-Ga_2_O_3_ material was about 4.4 eV, which effectively suppressed the complexation of photogenerated carriers, and a quantum yield of up to 36.1% for nitrogen fixation was obtained by the combined effect of in situ-grown CO_2_-induced electron transfer and photocatalyst surface electron transfer (Figure 2b). Meanwhile, methanol, ethanol, n-propanol, and n-butanol were employed as hole-trapping agents to further improve the conversion efficiency.

So far, metal oxides (mainly non-precious metal oxides) have attracted much attention because of their advantages such as easy synthesis, stability and control, low cost, and environmental friendliness. For example, SrTiO_3_, which is widely used in the field of water cracking, has also received some attention in photocatalytic ammonia synthesis. However, there is no universal consensus on the mechanism of photocatalytic reduction of N_2_ by metal oxides. Based on theoretical calculation, the dissociative mechanism and associative mechanism for nitrogen fixation have been gradually explored and tested. In recent years, some research results have provided new ideas and prospects for the application of metal oxides in photocatalytic NRR.

### 2.2. Metal Sulfides

**Figure 3:**
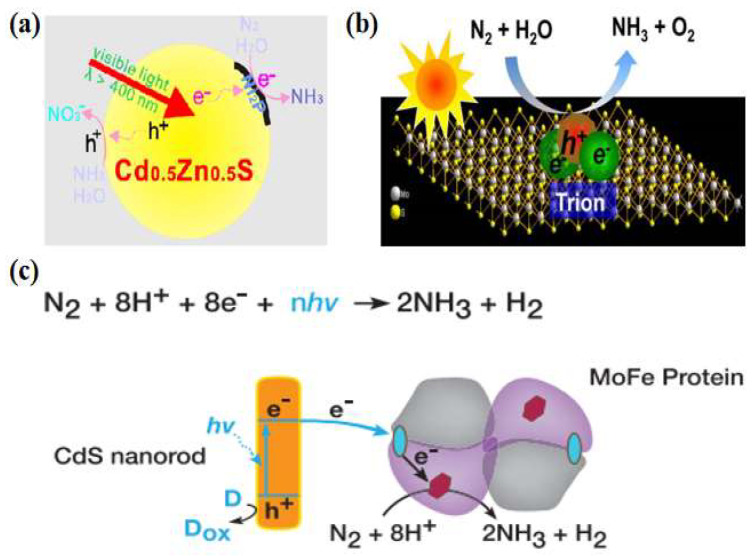
(**a**) Photocatalytic nitrogen fixation mechanism of Ni_2_P/Cd_0.5_Zn_0.5_S [31]; copyright 2017, Elsevier. (**b**) Schematic diagram of the exciton-induced multi-electron N_2_ reduction process [35]; copyright 2017, Elsevier. (**c**) Catalytic reaction mechanism of CdS: MoFe protein complexes [36]; copyright 2016, Elsevier.

Metal sulfides have excellent optical, electrical, and magnetic properties, and their narrow bandgap facilitates the absorption of visible light to obtain high light energy utilization. Khan et al. [30] used CdS/Pt/RuO_2_ composite to reduce N_2_ under visible light (λ = 505 nm) irradiation, and the activated dinitrogen reacted with [Ru(Hedta)(H_2_O)]^−^ to produce [Ru(Hedta)(N_2_)]^−^ complex. A continuous supply of photogenerated electrons from CdS to this complex reacts to form ammonia. As the photoreaction proceeded, the ammonia yield decreased due to photocorrosion by CdS. To improve the photocatalytic activity and stability, Ye et al. [31] used a Cd_0.5_Zn_0.5_S solid solution for photocatalytic nitrogen fixation for the first time and employed a transition metal phosphide (Ni_2_P) as a co-catalyst. Ni_2_P/Cd_0.5_Zn_0.5_S was used for photocatalytic nitrogen reduction reaction without adding any sacrificial agent. After irradiation with visible light (λ > 400 nm) for 1 h, the NH_3_ concentration reached 101.5 μmol·L^−1^. The quantum efficiency under 420 nm monochromatic light reached 4.32%, much higher than those of other semiconductors. As tested by time-resolved fluorescence spectroscopy, photocurrent, and electrochemical impedance spectroscopy, the samples with the addition of the co-catalysts rapidly transferred the photogenerated electrons to Ni_2_P through excellent heterogeneous interfacial contacts to reduce the charge complexation, thus improving the photogenerated carrier separation efficiency (Figure 3a). In addition, the photogenerated electron–hole pairs in the ultrathin transition metal sulfides (TMDs) could form tightly bound excitons, which give very high dissociation energies by trapping electrons. As a member of TMDs, MoS_2_ is getting much attention [32,33,34]. Sun et al. [35] found that ultrasonically treated ultrathin MoS_2_ could photocatalytically reduce nitrogen to ammonia with a photocatalytic ammonia yield of up to 325 μmol·g_cat_^−1^·h^−1^ without the use of a sacrificial agent or co-catalyst, and had considerable stability. Photogenerated excitons captured the free electrons in the ultrathin MoS_2_ to generate charged excitons near the Mo sites, which interacted with the adsorbed N_2_ to promote the multi-electron transfer, lower the reaction thermodynamic potential barrier, and accelerate the process of the photocatalytic reduction of nitrogen (Figure 3b).

Inspired by nitrogen-fixing enzymes, researchers have studied photocatalytic nitrogen reduction reactions in the cross-fertilized materials science and biology disciplines. Brown et al. [36] adsorbed MoFe proteins (the active site of nitrogen-fixing enzymes) onto CdS nanorods to form biological nanocomplexes and investigated their nitrogen-fixing activities. Photosensitization of the MoFe protein using CdS nanocrystals replaced ATP hydrolysis by capturing light energy (Figure 3c). Under visible light, the ammonia production rate reached 315 μmol·mg^−1^·min^−1^, which was on par with the biological nitrogen-fixing enzyme capacity. Given the prominent role of the MoFe factor in nitrogen-fixing enzymes, Banerjee et al. [37] deduced that solid compounds consisting of FeMoS inorganic clusters could reduce nitrogen in water to ammonia in the presence of light, and thus a combination of [Mo_2_Fe_6_S_8_(SPh)_3_]^3+^ and [Sn_2_S_6_]^4−^ clusters was used to constitute bionic sulfur compounds. The designed and synthesized Fe_2_Mo_6_S_8_ thiocolloid has strong light absorption, high specific surface area, and excellent water stability. Thus, its performance was superior to that of nitrogen-fixing enzymes. On this basis, Liu et al. [38] designed a novel thioglycolic system consisting of Fe_2_Mo_6_S_8_(SPh)_3_ and Fe_3_S_4_ mimetic clusters. The bonding between nitrogen and iron was determined using local orbital theory analysis, demonstrating that Fe was the active site for N_2_ binding and that it drives the nitrogen reduction reaction more readily than the Mo metal site [39,40].

Metal-sulfide-based photocatalysts have relatively narrow band gaps, abundant active sites, and adjustable electronic properties, which are suitable for nitrogen fixation. However, the metal sulfides applied to the photocatalytic reduction of N_2_ to date are mainly based on CdS, and the ammonia production rate is generally low. For other metal sulfides, such as two-dimensional metal disulfide and indium-based sulfide, the potential of catalytic nitrogen fixation has been preliminarily predicted by theory and experiment. Considering the diversity of metal sulfides, such catalysts need to be further explored as efficient artificial nitrogen fixation catalytic materials.

### 2.3. BiOX-Based Materials

Bismuth halide oxide (BiOX, X = Cl, Br, I) has attracted much attention due to its superior optical properties. Its layered structure provides ample space for atomic polarization and an internal electric field that facilitates the separation and transfer of photogenerated carriers [41,42,43]. The application of BiOX-based materials in photocatalytic nitrogen fixation has been demonstrated in recent works.

Li et al. [44] demonstrated that the photocatalytic reduction reaction of nitrogen could be realized under visible light without any organic sacrificial agent or precious metal co-catalyst using BiOBr nanosheets at room temperature and pressure. The prepared catalysts possessed electron-donating properties upon photoexcitation, driving the interfacial electron transfer from BiOBr nanosheets to adsorbed N_2_, and ammonia yields as high as 104.2 μmol·g_cat_^−1^·h^−1^ were obtained. Combined with theoretical simulations, the oxygen vacancies in BiOBr extend the activated N≡N bond length from 1.078 Å to 1.133 Å, promoting the activation of nitrogen molecules. Due to the generation of abundant oxygen vacancies on the surface, a defect state was formed at the bottom of the BiOBr conduction band, which inhibits the recombination of electron–hole pairs. In addition, the group examined the photocatalytic activity of BiOCl containing abundant oxygen vacancies [45]. The kinetics and mechanisms of the photocatalytic reactions differed due to the different exposed crystalline surfaces. The mechanism of nitrogen fixation on the (110) crystalline face follows a distal binding mechanism (N_2_ → •N-NH_3_ → •N + NH_3_ → 2NH_3_), while the reaction on the (010) face follows an alternating binding mechanism (N_2_ → N_2_H_3_ → N_2_H_4_). Under UV irradiation at a wavelength of 254 nm, the quantum yields of the BiOCl (001) and (010) crystal faces were 1.8% and 4.3%, respectively. To further demonstrate the effect of exposed crystal faces on photocatalytic activity, Bai et al. [46] prepared Bi_5_O_7_I nanosheets with different exposed crystal faces, in which the nitrogen fixation activities of the catalyst samples with exposed crystal faces of (001) and (100) were 111.5 mmol·L^−1^·h^−1^ and 47.6 mmol·L^−1^·h^−1^, respectively. The difference was due to the higher photogenerated carrier separation efficiency and more negative conduction band position (−1.45 eV) in Bi_5_O_7_I-001. Zeng et al. [47] successfully synthesized carbon-doped BiOI (C-BiOI) by hydrothermal reaction, demonstrating that the surface carbon elements adsorb nitrogen. The ammonia yield of C-BiOI-3 under visible light was as high as 311 μmol·g_cat_^−1^·h^−1^, about 3.7 times higher than that of pure BiOI. Carbon clusters entered the intercalation of BiOI crystals during the preparation process, interfered with the periodicity of the crystal lattice, and induced the generation of vacancies in the BiOI structure, which resulted in a decrease in the catalyst band gap and enhancement in visible light absorption, and the trapping of photogenerated electrons by the vacancies, which led to improvement in the charge separation efficiency and accelerated the photocatalytic reaction. In addition, carbon doping affected the morphology of the catalysts with reduced crystal size and increased specific surface area, facilitating the contact between the catalysts and reactants. However, the induced surface oxygen vacancies in BiOX-based materials were easily oxidized during the reaction process, decreasing photocatalytic NRR activity. To alleviate this difficulty, Wang et al. [48] designed ultrafine Bi_5_O_7_Br nanotubes with abundant sustainable oxygen vacancies to accelerate the photocatalytic reduction of nitrogen in aqueous solvent in order to synthesize ammonia without the addition of any sacrificial agents or co-catalysts. The synthesized sample has a large specific surface area (>96 m^2^·g^−1^), suitable light-absorbing band edges, and a continuous supply of surface oxygen vacancies, and thus the ammonia yield obtained is as high as 1.38 mmol·g_cat_^−1^·h^−1^, and the apparent quantum efficiency at 420 nm is close to 2.3%.

The indirect bandgap of BiOX material effectively hinders charge recombination. Its unique layered structure not only facilitates the generation of vacancies as active sites for catalytic reactions but also provides internal electric fields as driving forces for charge transfer. In addition, research was conducted on the photocatalytic reduction of N_2_ using BiOX substrate materials from the perspectives of defect engineering, surface engineering, and band gap structure adjustment. It is worth noting that high-quality 2D BiOX-based materials have a photocatalytic surface that changes with the progress of photo reactions and can serve as a dynamic crystal model for theoretical simulation. The combination of a dynamic simulation algorithm and experimental data can be used as a new simulation method to deeply understand the photocatalytic reaction mechanism.

### 2.4. Carbon-Based Materials

Carbon-based materials commonly used for photocatalysis include diamond, graphene, carbon nanotubes, and graphitic carbon nitride. Zhu et al. [49] prepared boron-doped diamonds to catalyze ammonia synthesis by nitrogen reduction under mild conditions. Transient absorption tests at a wavelength of 632 nm showed that diamond transfers solvated electrons to water when photoexcited. Comparative tests using samples and purchased product powders showed that the photocatalytic activity depended on the H terminals on the diamond surface and was correlated with the production of solvated electrons. In this catalytic process, the electrons were transported directly to the reactants without going through molecular adsorption on the catalyst’s surface, making it a new paradigm for photocatalytic reduction. Diamond’s stability and acid resistance set it apart from conventional photovoltaic materials. Bandy et al. [50] synthesized diamond thin films on Mo, Ni, and Ti metal substrates, and photoresponse tests showed that H-terminated thin films with a negative electron affinity drove nitrogen reduction.

**Figure 4:**
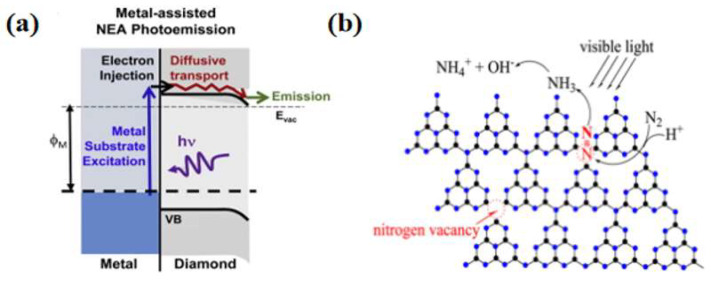
(**a**) Metal-assisted NEA photoelectron emission process [50]; copyright 2016, Elsevier. (**b**) Possible photocatalytic nitrogen fixation on M-GCN [56]; copyright 2016, Elsevier.

In contrast, O-terminated thin films showed almost no photocatalytic activity. The electrons in the metal substrate were transferred to the conduction band of the diamond through a barrier-free electron emission process, thus providing enough energy to participate in the nitrogen fixation reaction (Figure 4a). Graphene, as an allotrope of diamond with excellent electrical conductivity, is also considered an excellent substrate with the ability to activate N_2_. Tian et al. [51] demonstrated the ability of aluminum-doped graphene to convert nitrogen to ammonia through DFT simulations. Li et al. [52] proposed that FeN_3_-embedded graphene could be used as a raw material for photocatalytic nitrogen reduction through first-principle calculations. In addition, Perathoner et al. [53] used carbon nanotubes loaded with Fe as the photocatalyst to harvest an ammonia yield of 2.2 × 10^−3^ g·m^−2^·h^−2^ at ambient temperature and pressure. Liu et al. [54] prepared nitrogen-doped porous carbon (NPC) using pyrolysis of an imidazolium zeolite skeleton, which is a structure with high N content and tunable N species, to promote nitrogen molecule chemisorption and activation, thus addressing the problem of the slow kinetics of nitrogen fixation reactions.

The lack of active sites and photogenerated carriers in pure carbon materials limits their nitrogen fixation applications. Therefore, researchers have developed graphitic carbon nitride (g-C_3_N_4_)-based photocatalysts. Dong et al. [55] successfully synthesized g-C_3_N_4_ containing nitrogen vacancies by nitrogen heat treatment and reported the effect of nitrogen vacancies on the activity of semiconductor photocatalytic nitrogen reduction reactions. In the photocatalytic experiments, it was observed that the nitrogen vacancies endowed the g-C_3_N_4_ with photocatalytic nitrogen fixation ability. Since nitrogen vacancies have the same shape and size as nitrogen atoms, they could selectively adsorb activated nitrogen, and thus the photocatalytic nitrogen fixation process did not interfere with other gases. In addition to this advantage, nitrogen vacancies improve the separation efficiency of photogenerated carriers and promote the transfer of photogenerated electrons from g-C_3_N_4_ to adsorbed N_2_. Wu et al. [56] prepared a spongy g-C_3_N_4_, whose excellent nitrogen fixation capability benefited from the trapping of photogenerated electrons by the surface nitrogen vacancies (Figure 4b). Cao et al. [57] used urea as the raw material and, using a simple one-step separation method, synthesized amine-functionalized ultrathin g-C_3_N_4_ nanosheets. Compared with bulk g-C_3_N_4_, the synthesized g-C_3_N_4_ nanosheets have a larger specific surface area, higher reduction potential and carrier separation efficiency, and improved photocatalytic activity and stability of nitrogen fixation reaction under visible light irradiation.

Li et al. [58] pretreated the samples with alkali solutions of appropriate concentrations, which resulted in the C=N bond breaking and surface K^+^ grafting of the homotriazine structural unit in g-C_3_N_4_. The g-C_3_N_4_ etched with KOH solution was used as the photocatalytic material, while methanol solution was introduced as the proton source for the first time. The photocatalyst showed an ammonia yield of 3.632 mmol·g^−1^·h^−1^ and an apparent quantum efficiency of up to 21.5% under light irradiation with a wavelength of ~420 nm. The enhancement in the catalytic activity was due to the combined effects of various aspects, including the enhanced absorption of light, the fast dissolution of N_2_ in methanol solution, the increase in active centers on the catalyst surface, and the promotion of carrier transfer and ammonia desorption by CH_3_OH and surface K^+^. In addition to structural optimization, strategies such as elemental doping and material composites have also been used as modification methods for g-C_3_N_4_. For example, Hu et al. [59] synthesized honeycomb-shaped Fe-doped g-C_3_N_4_ by controlling the concentration of Fe^3+^. Fe^3+^ enters the lattice sites and stably exists in the structure of g-C_3_N_4_ through Fe-N coordination bonding. The Fe metal sites became the active centers for the adsorption and activation of nitrogen molecules and promoted the interfacial charge transfer between the catalyst and the nitrogen molecules, significantly improving nitrogen fixation capacity. The best ammonia yield was obtained for Fe0.05-CN, about 5.40 mg·L^−1^·h^−1^·g_cat_^−1^, close to 13.5 times that of pure g-C_3_N_4_. In addition, the photocatalytic nitrogen fixation performance of g-C_3_N_4_ semiconductor-based composites, such as MnO_2-x_/g-C_3_N_4_, Ti_3_C_2_/g-C_3_N_4_, and g-C_3_N_4_/FeOCl, was significantly improved due to the construction of heterostructures to promote the separation and transfer of photogenerated carriers [60,61].

At present, carbon-based materials applied in the photocatalytic reduction of N_2_ are mainly g-C_3_N_4_ and 2D graphene. Due to their unique structure, excellent light absorption performance, and conductivity, both are considered nitrogen-fixing photocatalysts with infinite potential. In addition, inspired by the size-dependent photoluminescence effect, modification of carbon-based materials by morphological regulation is an effective way to improve the photoactivity of catalysts. For example, the design of zero-dimensional carbon quantum dots enables carbon-based materials to obtain the advantages of adjustable chemical structure, high quantum efficiency, and good biocompatibility, so as to improve catalyst reaction activity.

### 2.5. MOFs and Derivatives

Metal-organic skeletons (MOFs), as porous materials with high crystallinity and tunable organic ligands, have been shown to have efficient photocatalytic activity. In recent years, researchers have designed a series of MOFs and their derivatives and performed photocatalytic nitrogen reduction experiments [62,63,64,65].

**Figure 5:**
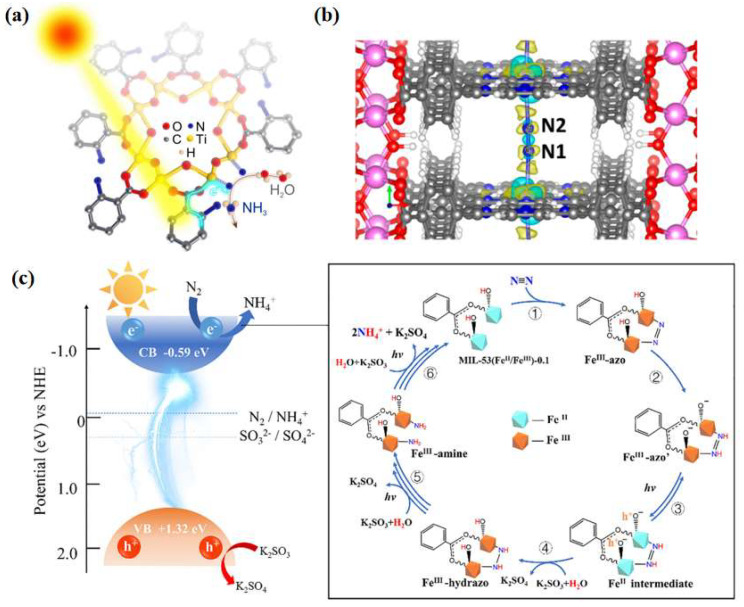
(**a**) Possible mechanism of NH_2_-MIL-125(Ti) photocatalytic immobilization of N_2_ [66]; copyright 2020, Elsevier. (**b**) Charge difference diagram of N_2_ adsorbed on AlPMOF (Fe): yellow, positive density difference; cyan, negative density difference [67]. (**c**) Mechanism of photocatalytic N_2_ reduction by MIL-53 (Fe^II^/Fe^III^) in visible light [70]. Copyright 2020, Elsevier.

Huang et al. [66] prepared NH_2_-MIL-125(Ti) catalysts by integrating metal sites with amine-based functional groups and applied them to photocatalytic nitrogen fixation reaction at ambient temperature and pressure, obtaining an ammonia yield of 12.3 μmol·g^−1^·h^−1^ under visible light. Through ligand functionalization, the light absorption range of the MOF materials was extended to the visible light region. Simultaneous electron transfer between the ligand and the metal-induced Ti^3+^ production provides abundant active sites for nitrogen reduction (Figure 5a). Inspired by chlorophyll, Shang et al. [67] developed a porphyrin-based metal-organic skeleton (PMOF) with Fe as the active center, with Al characterized as the metal node with excellent stability, and Fe atoms dispersed on each porphyrin ring to facilitate nitrogen adsorption activation. Calculations showed that the Fe-N site in Al-PMOF(Fe) acts as the active center of the photocatalytic reaction and reduces the difficulty of the rate-determining step in the reaction process (Figure 5b). The ammonia yield of Al-PMOF(Fe) was 127 μg·g^−1^·h^−1^, which was a 50% improvement in performance compared to the pristine Al-PMOF catalysts. Zhang et al. [68] simulated the π-orbitals of the reverse feeding mechanism of a designed and synthesized MOF-76(Ce) material, in which Ce acts as the active center for capturing photogenerated electrons. Experimental results and theoretical analyses showed that the presence of cerium metal in a ligand-unsaturated state (Ce-CUS) on the surface of MOF-76(Ce) nanorods could provide unoccupied 4f orbitals to collect electrons and transfer them to N_2_. The synthesized materials exhibited excellent photocatalytic nitrogen reduction performance with ammonia yields as high as 34 μmol·g^−1^·h^−1^ at ambient temperature and pressure. Xu et al. [69] successfully synthesized CeZr_5_-MOF(UiO-66) bimetallic photocatalysts using the rare earth element Ce to partially replace Zr. Ce was introduced into the nodes of Zr-MOFs(UiO-66) to form CeZr_5_ clusters, which enhanced the separation and transfer rate of the photogenerated electron–hole pairs through the charge-transfer process between the ligand and the metal, thus enhancing the photocatalytic nitrogen fixation activity. In addition, the photocatalytic performance was increased linearly with the increase in Ce content when the Ce content was lower than 20%. The photocatalytic nitrogen fixation activity was 200.13 μmol·g^−1^·h^−1^, 105.9% higher than that of Zr-UiO-66. Zhao et al. [70] designed a MOF-based material MIL-53 (Fe^II^/Fe^III^), in which Fe^II^ and Fe^III^ constituted a mixed-valence metal cluster, which mimicked the Fe^2+^ active site and the high-valence metal ions in nitrogen fixation enzymes, respectively. The Fe^II^/Fe^III^ ratio was crucial for coordinating the catalytic activity and the stability of the backbone structure, and the experimentally obtained optimal Fe^II^/Fe^III^ ratio was 1.06:1, which gives the highest ammonia yield of 306 μmol·h^−1^·g^−1^. The activity enhancement of the MIL-53(Fe^II^/Fe^III^) material was attributed to the combined effect between catalytic and non-catalytic functions, i.e., increased ligand-unsaturated active sites, prolonged visible absorption edge (650 nm), and reduced photogenerated carrier complexation rate (Figure 5c).

Introducing foreign atoms into the main lattice of a semiconductor induces defective states in the electronic and chemical structure, which in turn affects the overall performance of the catalyst [71]. In the photocatalytic nitrogen fixation process, the critical roles of the dopant sites were to act as active centers for N_2_ adsorption activation and to promote photogenerated charge separation. In addition to the materials mentioned above, Table 2 organizes the recent representative photocatalysts for nitrogen fixation and summarizes the photocatalytic systems by catalyst type, sacrificial agent, light source, and ammonia yield.

## 3. Other Photocatalytic Nitrogen Fixation Materials

In addition to the common photocatalytic materials mentioned above, single-atom catalysts, black phosphorus, layered double hydroxides, molecular sieves, and plasmonic materials have also been shown to have photocatalytic nitrogen fixation activity.

The size of the catalyst directly affects the number of surface low coordination sites, influencing the binding strength to the reactants and determining the catalytic performance to a certain extent. Single-atom metals dispersed on the carrier have the characteristics of uniform catalytic active sites, the low coordination environment of metal atoms, and optimal metal utilization efficiency. Hence, single-atom catalysts have outstanding catalytic activity, stability, and selectivity and have recently attracted wide attention [101]. Liu et al. [102] designed and prepared Ru single-atom modified oxygen-rich vacancy TiO_2_ nanosheets, which catalyzed the nitrogen under xenon lamp light reduction to ammonia. The composite photocatalyst containing 1 wt% Ru showed a significantly improved NH_3_ generation rate of 56.3 μg·h^−1^·g_cat_^−1^, two times higher than the performance of the pure TiO_2_ nanosheets. DFT calculations showed that the single Ru metal atoms were immobilized on oxygen vacancies, which inhibited the hydrogen precipitation reaction, facilitated the chemical adsorption of N_2_, and improved the carrier separation process, resulting in the enhancement of the photocatalytic reduction ability.

Layered double hydroxides (LDHs) belong to two-dimensional nanomaterials, which provide new resources for developing novel catalytic and photocatalytic materials due to their controllable particle size, flexible composition, and easy synthesis. Zhang et al. [103] successfully synthesized ultrathin nanosheets of ZnAl-LDH by a facile co-precipitation method. The 0.5%-ZnAl-LDH nanosheets (Cu doped with 0.5 mol%) with abundant oxygen vacancies and electron-rich ligand unsaturated Cu^δ+^ exhibited excellent photocatalytic activity and stability under UV–vis irradiation. A catalytic reaction rate of 110 μmol·g^−1^·h^−1^ (4.12 μmol·m^−2^·h^−1^) was achieved at ambient temperature and pressure without any sacrificial agent or co-catalyst addition. Detailed structural analyses and density-functional theory calculations indicate that the oxygen vacancies and Cu^δ+^ in 0.5%-ZnAl-LDH contribute to the efficient separation and transfer of photogenerated electrons and holes, activating nitrogen molecules and accelerating the multi-electron reduction process.

Plasma catalysis originates from local surface plasmon resonance of metal nanostructures and has been proven to be an effective method for converting light energy into chemical energy. Thanks to the surface plasmon resonance effect of plasma metals and the Schottky barrier formed at the interface with semiconductors, loading plasma metals (Au, Ag, Cu) on semiconductors can effectively expand the light absorption of catalysts to the visible light region and improve the separation efficiency of photogenerated carriers [104]. Xiong’s team [105] selected Au nanocrystals to absorb light, and Ru atoms to adsorb N_2_ molecules as active sites. They reported a surface plasma that can provide sufficient energy to activate N_2_ through a dissociation mechanism in the presence of water and incident light. This mechanism was demonstrated using in situ synchrotron radiation infrared spectroscopy and near-ambient pressure X-ray photoelectron spectroscopy. The photocatalytic nitrogen fixation reaction was carried out using AuRu core-antenna nanostructures with a wide light absorption range and a large number of active sites at room temperature, two atmospheres, and without any sacrificial agents, resulting in an ammonia generation rate of 101.4 μmol·g^−1^·h^−1^. Theoretical simulations have verified that the electric field enhanced by surface plasma, plasma hot electrons, and interface hybridization may play a key role in N≡N dissociation. This work demonstrates the importance of surface plasma in activating inert molecules.

## 4. Conclusions and Prospects

In the future, the preparation of photocatalysts could be approached by taking into account the following aspects:

In view of the conformational relationship between morphology, structure, and performance, a structural morphology with a large specific surface area could be prepared by changing the reaction conditions, composition, and ratio of precursors to improve the contact probability between the catalysts and the reactants, increase the surface active sites, and promote the adsorption and activation of N_2_.

By introducing vacancies, constructing heterojunctions, and element doping, the band gap bandwidth of the semiconductor could be modulated, resulting in enhanced catalyst response to visible light and improved light energy utilization.

To extend the lifetime of photogenerated carriers and to improve the quantum efficiency of photocatalytic reactions, a modification strategy may be utilized to improve the separation and transport efficiency of photogenerated electrons and holes in catalysts. We analyze the mechanisms of photocatalysts and reactions to achieve high energy utilization and excellent catalytic activity in the nitrogen fixation process. We strive to create a reasonable and efficient photocatalytic reaction system tailored for nitrogen fixation.

## Figures and Tables

**Table 1:** Comparison between the Haber–Bosh process and photocatalytic nitrogen fixation process.

	Haber–Bosh	Photocatalytic Nitrogen Fixation
Reaction equation	N_2_ + 3H_2_ → 2NH_3_	2N_2_ + 6H_2_O → 4NH_3_ + 3O_2_
Hydrogen source	Natural gas	Water
Catalysts	Fe/Ru-based catalysts	Mainly semiconductors
Temperature	400–600 °C	Room temperature
Pressure	150–300 atm	1 atm
Energy source	Fossil fuel	Solar energy

**Table 2:** Recently reported photocatalytic systems for nitrogen fixation.

Type	Photocatalyst	Sacrificial Agent	Light Source	Ammonia Yield/μmol·g_cat_^−1^·h^−1^	Ref.
Metal oxides	N-TiO_2_	-	300 W Xe lamp (λ > 400 nm)	80.09	[72]
	Ni-TiO_2_	-	300 W Xe lamp	46.8	[73]
	Rutile TiO_2_	Methanol	300 W Xe lamp (λ > 420 nm)	116	[74]
	Fe-TiO_2_-SiO_2_	-	300 W Xe lamp	32	[75]
	TiO_2_-Au-BiOI	-	300 W Xe lamp	534.5	[76]
	Cu_x_O/CNNTs	Ethanol	300 W Xe lamp (λ > 420 nm)	1380	[77]
	WO_3_/B-CN	Methanol	300 W Xe lamp (λ > 420 nm)	450.94	[78]
	U-Cu_2_O-0.05 M-2 h	-	300 W Xe lamp (λ > 400 nm)	4100	[79]
	Cu_2_O/MoS_2_/ZnO-cm	-	350 W Xe lamp (λ > 420 nm)	111.94	[80]
Metal sulfides	1T’-MoS_2_/CNNC	Methanol	300 W Xe lamp (AM 1.5G filter)	9800	[81]
	CdS/CNS	-	350 W Xe lamp (400–800 nm)	327	[82]
	5%NiS-KNbO_3_	Ethanol	300 W Xe lamp	155.6	[83]
	MoS_2_/OPC	-	300 W Xe lamp (λ > 400 nm)	37.878	[84]
	Zn_0.8_Cd_0.2_S	Sodium sulfite	300 W Xe lamp (λ > 420 nm)	66.91	[85]
	Fe-MoS_y_/Cu_x_S	-	350 W Xe lamp (λ > 420 nm)	8171	[86]
BiOX-based materials	BiVO_4_	-	300 W Xe lamp (200–800 nm)	103.4	[87]
	P-Bi_2_WO_6_	-	300 W Xe lamp	73.6	[88]
	Bi@BiOBr-Bi_2_MoO_6_	-	300 W Xe lamp	167.2	[89]
	Fe-BiOCl	-	300 W Xe lamp (200–800 nm)	60	[90]
	Mo-Bi_5_O_7_Br-1	-	300 W Xe lamp (λ > 420 nm)	122.9	[91]
	Bi_5_O_7_Br	-	300 W Xe lamp (λ > 400 nm)	12700	[92]
Carbon-based materials	S-CNNTs	Ethanol	300 W Xe lamp (λ > 420 nm)	640	[93]
	B-C_3_N_5_	Methanol	300 W Xe lamp (200–2500 nm)	421.18	[94]
	Ti_3_C_2_/g-C_3_N_4_	Methanol	300 W Xe lamp (λ > 420 nm)	601	[95]
	RuPd NPs/C_3_N_4_	Ethanol	300 W Xe lamp (λ > 420 nm)	1389.84	[96]
	NYF(15)/NV-CNNTs	Ethanol	300 W Xe lamp (λ > 420 nm)	1720	[97]
MOF-based materials	Zn-MIL-88A	-	300 W Xe lamp	300	[98]
	Au@UiO-66/PTFE membrane	-	300 W Xe lamp (λ > 400 nm)	360	[99]
	MOF@DF-C_3_N_4_	Methanol	300 W Xe lamp (λ > 400 nm)	2320	[100]

## Data Availability

Not applicable.

## Sample Availability

Samples of the compounds are available from the authors.
